# Ethanol modulation of mammalian BK channels in excitable tissues: molecular targets and their possible contribution to alcohol-induced altered behavior

**DOI:** 10.3389/fphys.2014.00466

**Published:** 2014-12-02

**Authors:** Alex M. Dopico, Anna N. Bukiya, Gilles E. Martin

**Affiliations:** ^1^Department of Pharmacology, College of Medicine, The University of Tennessee Health Science CenterMemphis, TN, USA; ^2^Department of Psychiatry, The University of Massachusetts Medical SchoolWorcester, MA, USA

**Keywords:** slo1 proteins, BK beta subunits, membrane lipids, ethanol-recognition site, n-alkanols, alcohol tolerance, ion channels

## Abstract

In most tissues, the function of Ca^2+^- and voltage-gated K^+^ (BK) channels is modified in response to ethanol concentrations reached in human blood during alcohol intoxication. In general, modification of BK current from ethanol-naïve preparations in response to brief ethanol exposure results from changes in channel open probability without modification of unitary conductance or change in BK protein levels in the membrane. Protracted and/or repeated ethanol exposure, however, may evoke changes in BK expression. The final ethanol effect on BK open probability leading to either BK current potentiation or BK current reduction is determined by an orchestration of molecular factors, including levels of activating ligand (Ca^2+^_i_), BK subunit composition and post-translational modifications, and the channel's lipid microenvironment. These factors seem to allosterically regulate a direct interaction between ethanol and a recognition pocket of discrete dimensions recently mapped to the channel-forming (slo1) subunit. Type of ethanol exposure also plays a role in the final BK response to the drug: in several central nervous system regions (e.g., striatum, primary sensory neurons, and supraoptic nucleus), acute exposure to ethanol reduces neuronal excitability by enhancing BK activity. In contrast, protracted or repetitive ethanol administration may alter BK subunit composition and membrane expression, rendering the BK complex insensitive to further ethanol exposure. In neurohypophyseal axon terminals, ethanol potentiation of BK channel activity leads to a reduction in neuropeptide release. In vascular smooth muscle, however, ethanol inhibition of BK current leads to cell contraction and vascular constriction.

## Introduction

Ca^2+^-activated K^+^ channels are defined by their high selectivity for K^+^ over other monovalents and enhanced activity upon increases in intracellular Ca^2+^ (Ca^2+^_i_). Based on unitary conductance (γ), Ca^2+^-activated K^+^ channels have been classified into large (BK), intermediate (IK) and small conductance (SK) channels. These phenotypes also present differential sensitivity to Ca^2+^_i_, membrane voltage and distinct peptide blockers (Latorre et al., 1989; Stocker, 2004; Salkoff et al., 2006). The term BK channel, however, more properly applies to not only Ca^2+^-activated K^+^ channels of large conductance, i.e., BK α subunits or slo1 channels, which are products of the *Slo1, KCNMA1* gene or its orthologs (see Table 1 for nomenclature), but also to the channel-forming protein products of *Slo2* and *Slo3*, which are primarily gated not by Ca^2+^_i_ but by Na^+^/Cl^−^ and H^+^, respectively (Schreiber et al., 1998; Dryer, 2003; Xia et al., 2004; Salkoff et al., 2006). In this mini-review, however, we will use “BK channel” to designate a functional channel that results from tetrameric association of slo1 proteins and may include additional regulatory subunits that contribute to the current phenotype (see below).

**Table 1:** **Nomenclature of large-conductance K^+^ channel proteins and genes used in this review**.

BK	Protein complex forming an ion channel with a phenotype that combines high-conductance for K^+^ with voltage- and Ca^2+^-gating, disregarding subunit composition. Also cited in literature as BK_Ca_ or MaxiK channels.
*KCNMA1*	Mammalian gene that encodes the BK channel-forming slo1 protein, so called BK α subunit.
*KCNMB1-4*	Genes that encode the regulatory BK β subunits, these subunits being unable to form functional channels by themselves. Four β subunits have been identified (β1-4), each of the four types resulting from its corresponding gene (*KCNMB1-4*).
*Slo1*	Same as *KCNMA1*.
*Slo*	General term to define any non-mammalian ortholog of *Slo1*.
xslo1	BK channel-forming α subunit, where “x” denotes the species of origin (e.g., hslo1 from human, mslo1 from mouse, etc.) For consistency with previously published work, an exception was made for cbv1, which denotes slo1 proteins cloned from rat cerebral blood vessel (artery) myocytes.
slo2	High-conductance K^+^ channel protein gated by Na^+^_i_ or Cl^−^_i_.
slo3	High-conductance for K^+^ channel protein gated by H^+^_i_/OH^−^_i_.

In most neurons, K^+^ efflux due to Ca^2+^-activated K^+^ channel activity effectively drives the membrane potential toward more negative values leading to reduced excitability (Wong and Prince, 1981; MacDermott and Weight, 1982; Brown et al., 1983; Sah, 1996). This led to the early speculation that Ca^2+^-activated K^+^ “conductances” could be modified by alcohols and other sedative/hypnotic agents in central nervous system (CNS) neurons (Krnjevic, 1972; Nicoll and Madison, 1982). Early studies on modulation of Ca^2+^-activated K^+^ currents by ethanol were conducted on non-neuronal preparations and/or using alcohol concentrations well above circulating ethanol levels that are usually lethal in alcohol-naïve mammals (>100 mM), as reviewed elsewhere (Dopico et al., 1999a). A few early studies, however, did show that 5–20 mM ethanol (legal intoxication in the US is defined by 10–17.4 mM ethanol in blood) applied to hippocampal CA1 or CA3 neurons and granule cells, and cerebellar Purkinje cells enhanced a Ca^2+^-dependent after-hyperpolarization while increasing overall K^+^ conductance (Carlen et al., 1982, 1985; Niesen et al., 1988). Likewise, ethanol concentrations as low as 5 mM activate a Ca^2+^-activated K^+^ conductance in *Helix aspersa* right parietal ganglion (Madsen and Edeson, 1990). From these early studies, however, it was not possible to discern the Ca^2+^-activated K^+^ channel type affected by ethanol. In addition, these and later studies conducted in intact cells could not address whether ethanol effect on Ca^2+^-activated K^+^ current resulted from drug action on the Ca^2+^-activated K^+^ current itself or, rather, was secondary to ethanol modulation of Ca^2+^-sources that controlled Ca^2+^_i_-activated K^+^ channel activity.

BK channels received particular attention as functional targets of ethanol in the CNS as they are usually expressed and play major roles in all three neuronal compartments: somata, axon terminals and dendrites. Moreover, the channel's sensitivity to both voltage and Ca^2+^_i_ places it at the nexus of many cellular pathways associated with neuronal plasticity. BK channel pluripotency is further underscored by a recent study showing its presence in the neuronal nuclear membrane where it controls Ca^2+^ flux and gene expression (Li et al., 2014). At the presynaptic membrane, BK channels control the release of neurotransmitters by dampening the depolarization evoked by incoming action potentials (APs) (Raffaelli et al., 2004; Wang, 2008). On the post-synaptic side, BK channels contribute to AP shaping (Faber and Sah, 2002, 2003) and patterning (Jin et al., 2000; Zhang et al., 2003; Brenner et al., 2005; Meredith et al., 2006), and modulate α-amino-3-hydroxy-5-methyl-4-isoxazolepropionic acid (AMPA)- and N-methyl-D-aspartic acid (NMDA)-mediated excitatory post-synaptic potentials (EPSPs) (Isaacson and Murphy, 2001; Liu et al., 2011). The BK channel also controls dendritic excitability (Golding et al., 1999; Wessel et al., 1999; Rancz and Häusser, 2006; Benhassine and Berger, 2009), as well as retrograde propagation of somatic APs to the dendrites (Wessel et al., 1999; Ji and Martin, 2012).

By the mid to late nineties, using isolated neurohypophyseal axon terminals and pituitary epithelial-like tumor cell lines (GH3 cells) from the rat, two groups communicated the selective activation of BK channels by acute exposure to clinically relevant ethanol concentrations: half-maximal effective concentration (EC_50_) ≈ 22 mM; maximal effective concentration (EC_max_) ≤ 100 mM (Dopico et al., 1996; Jakab et al., 1997). Experimental conditions from these two studies demonstrated that ethanol action was due to drug targeting of the BK channel complex itself and/or its immediate proteolipid environment. Since then, activation of native BK channels by brief exposure to clinically relevant ethanol levels has been extended to both excitable and non-excitable tissues (Brodie et al., 2007; Martin et al., 2008; Pietrzykowski et al., 2008; Bukiya et al., 2009; Wynne et al., 2009; Velázquez-Marrero et al., 2011; Bettinger et al., 2012; Handlechner et al., 2013; Liu et al., 2013; Davis et al., 2014; Malysz et al., 2014). In parallel, several groups have documented ethanol-SK channel functional interactions and their relevance to alcohol-induced modified behaviors. Literature on ethanol and SK channels has been reviewed elsewhere (Brodie et al., 2007; Mulholland et al., 2009) and is not dealt with in this review, which focuses on modulation of BK channels from mammalian systems in response to acute ethanol administration. In particular, we concentrate on the many molecular entities and mechanisms that determine the final BK current response to brief (acute) ethanol exposure, and the consequences of such modulation on the physiology of excitable tissues. Neuronal and behavioral adaptations involving BK channels or neuronally-expressed genes coding for BK channel subunits to repetitive or protracted ethanol exposure have been well documented in both mammals and non-mammals (Mulholland et al., 2009; Treistman and Martin, 2009; McIntire, 2010; Ghezzi and Atkinson, 2011) and comprehensively treated in this volume by Bettinger and Davies (2014).

## Ethanol effect on BK currents in alcohol-naïve systems: phenomenology and modifications in channel gating

Following brief exposure (<5 min) of native BK channels to clinically relevant ethanol concentrations (10–100 mM), steady-state ionic current potentiation, refractoriness and reduction have all been observed, this heterogeneity being reported even between different compartments of a same neuronal type (Dopico et al., 1999b; Martin et al., 2004; Wynne et al., 2009). The variety of molecular factors that contribute to such heterogeneity are extensively discussed in a separate section below. However, some generalizations from studies of acute ethanol action on native channels and recombinant BK proteins expressed in natural membranes or following channel reconstitution into artificial planar bilayers can be made. In the vast majority of cases, provided that the channel consists of homomeric slo1 or heteromeric slo1 + β4 subunits and is evaluated at Ca^2+^_i_ within nM to low μM, a few min exposure to ethanol potentiates steady-state current (Brodie et al., 2007; Mulholland et al., 2009). This potentiation occurs in absence of changes in K^+^ permeability (Dopico et al., 1996, 1998; Jakab et al., 1997; Gruß et al., 2001; Martin et al., 2004) or selectivity over Na^+^ (Dopico et al., 1996, 1998) and BK membrane expression (Dopico et al., 1998) but results from ethanol-induced increase in channel open probability (Po). In neurohypophyseal terminals, this increase is consistently observed provided that alcohol-naïve preparations are briefly exposed to the drug (a few min) (Dopico et al., 1996) and totally disappears after 12 min of constant ethanol exposure (Pietrzykowski et al., 2004). The mechanisms leading to this rapid desensitization to ethanol exposure remain to be fully addressed (see next Sections on Molecular Targets). However, when neurohypophyseal explants are subject to 24 h-long ethanol exposure, decreased BK current density has been linked to a reduction in BK channel clustering in the cell membrane and internalization of the channel α (slo1) subunit (Pietrzykowski et al., 2004).

Following brief exposure to alcohol-naïve systems, ethanol-induced maximal increase in BK is reached at 75–100 mM, with an EC_50_ = 20–25 mM (reviewed in Brodie et al., 2007), the latter being close to blood alcohol levels considered legal intoxication in most US states (0.08g/dl = 17.4 mM) (Diamond, 1992; Thombs et al., 2003). While these ethanol concentrations are significantly higher than those of other BK channel modulators (Weiger et al., 2002), different studies ruled out that an osmotic load to the membrane and/or channel complex was a major contributor to ethanol action on BK currents (Dopico et al., 1996, 1998; Jakab et al., 1997). Because ethanol acute action on BK channels studied in cell-free systems like isolated membrane patches or following reconstitution into planar lipid bilayers of simple composition mimics drug action in intact cells (reviewed in Brodie et al., 2007; Mulholland et al., 2009), it is possible to conclude that acute ethanol modulation of BK current in alcohol-naïve systems is largely independent of the continuous presence of cytosolic signals, internal organelles, complex membrane cytoarchitecture, and ethanol metabolism. It should be noted, however, that acetaldehyde applied to the intracellular surface of GH3 cell membrane patches was able to reduce ethanol-induced activation of BK channels (Handlechner et al., 2013), raising the hypothesis that an ethanol metabolite in excitable tissues contributes to the overall effect of ethanol on native BK currents.

Increased BK Po by ethanol itself results from several modifications in both open and closed-times distributions that lead to minor increase in mean open time and major decrease in mean closed time, the latter primarily due to drug-induced destabilization of channel long-closed states (Dopico et al., 1996, 1998; Chu et al., 1998; Crowley et al., 2003). A 10-state model of slo1 (mslo1, from mouse brain; mbr5 variant) channel gating reveals that ethanol modifies Ca^2+^-dependent parameters, such as the channel open conformation-Ca^2+^ dissociation (K_O_) and closed conformation-Ca^2+^ dissociation (K_C_) constants. In contrast, Ca^2+^-independent parameters, such the equivalent gating charge associated with the open-to-closed equilibrium (Q) and the open-to-closed equilibrium constant in absence of Ca^2+^ and transmembrane voltage (L_0_ or “intrinsic gating”) remain unchanged. Moreover, slo1 becomes ethanol-resistant when gated by voltage/Mg^2+^_i_ in absence of activating Ca^2+^_i_, with fully effective activatory concentrations of ethanol (100 mM) failing to modify mslo1 Po. Consistently, combination of amino acid substitutions (5D5N) in the Ca^2+^-bowl and in the high affinity regulator of conductance for K^+^ (RCK) 1 domain (D362A, D367A), which render both high affinity Ca^2+^_i_-recognition sites non-functional, results in a channel that is ethanol-resistant. However, substitutions that hamper each site result in slo1 channels that retain ethanol sensitivity. These data indicate that ethanol action on BK channels requires activating Ca^2+^_i_. Moreover, as far as Ca^2+^_i_ is able to interact with one of its physiological recognition sites, the BK channel is activated by ethanol (Liu et al., 2008). The structural basis of ethanol activation of slo1 channels and its relation to Ca^2+^_i_ is provided in a separate section.

The Ca^2+^_i_-dependence of ethanol action, however, further conditions drug action on slo1 channels: ethanol potentiation of Po and macroscopic current progressively diminishes as Ca^2+^_i_ increases until ethanol becomes an inhibitor of BK activity; for homomeric slo1 channels, whether mslo mbr5 or cbv1 (from rat cerebral artery myocytes), the “cross-over” from ethanol-induced activation to ethanol-induced inhibition occurs at ~20 μM Ca^2+^_i_ (Liu et al., 2008; Bukiya et al., 2009). Remarkably, this cross-over can be shifted by modulators that fine-tune the overall Ca^2+^_i_ sensitivity of the native BK channel, accessory β1 subunits in particular (see separate section). An empirically-derived single channel kinetic model reveals that ethanol-induced inhibition of slo1 Po is related to the drug-induced facilitation of channel dwelling into Ca^2+^_i_-driven low Po modes (Liu et al., 2008), an action that can be conceptualized into ethanol-induced facilitation of Ca^2+^_i_-driven BK channel “desensitization” (Dopico and Lovinger, 2009).

In synthesis, exposure of BK channels to clinically-relevant ethanol concentrations in alcohol naïve, excitable cells under physiological, resting conditions usually results in BK current potentiation, yet refractoriness or inhibition may occur. This heterogeneous response is determined by several molecular entities, which are individually discussed in separate sections below. Ethanol action on BK ionic current results from modification of Po, this action being dependent on the ion that activates the channel under physiological conditions, that is, Ca^2+^_i_.

## Changes in physiology or behavior related to modification of BK currents by acute ethanol exposure

Regulation of BK Po and thus, steady-state ionic current by ethanol exposure has been implicated in alcohol-induced modification of physiology and behavior (reviewed in Brodie et al., 2007; Mulholland et al., 2009; Treistman and Martin, 2009; McIntire, 2010; Ghezzi and Atkinson, 2011). Early studies concentrated in neurosecretory cells given the central role of BK channels in controlling AP firing and hormone/neurotransmitter release (see above). In rats, ethanol-induced potentiation of BK currents, together with drug-induced inhibition of voltage-dependent Ca^2+^ channels (Wang et al., 1994) has been recognized as a central mechanism in ethanol-induced inhibition of vasopressin and oxytocin release by supraoptic axon terminals (Dopico et al., 1995; Knott et al., 2002). Likewise, ethanol-induced BK channel activation in GH3 and GH4/C1 rat pituitary tumor cells would likely lead to inhibition of hormone release by alcohol (Jakab et al., 1997, 2006). In spite of BK current potentiation, ethanol actually increases growth hormone secretion by GH3-GH4/C1 cells, which has been attributed to increased Ca^2+^_i_ (Stojilkovic et al., 2005; Jakab et al., 2006; Brodie et al., 2007) and to cell swelling itself being able to evoke hormone release (Strbak, 2006). Indeed, ethanol has been proven to increase Ca^2+^_i_ and cause cell swelling in GH3-GH4/C1 cells (Jakab et al., 2006).

In the rat and mouse striatum, ethanol potentiation of BK currents has been demonstrated to reduce nucleus accumbens medium spiny neurons (MSN) AP firing rate and thus, decrease neuronal excitability (Martin et al., 2004, 2008), the consequences of this ethanol action being linked to ethanol-induced perturbation of motor behavior and alcohol preference (see below and also review by Treistman and Martin, 2009). A decrease in AP frequency in response to 40 mM ethanol has been reported in dorsal root ganglia (DRG) neurons that show positive staining for isolectin B4, a marker for nociceptive neurons. Ethanol also shortens AP duration and increases AP mean threshold, these ethanol actions being blunted by selective blockade of BK channels by iberiotoxin (Gruß et al., 2001). Thus, authors of this study proposed that ethanol actions leading to reduced firing activity and decreased excitability of distinct DRG neurons might contribute to ethanol's analgesic effect in the peripheral nervous system.

In *Caenorhabditis elegans*, ethanol activates BK channels *in vivo*. Notably, the behavioral phenotype of slo1 gain-of-function mutants resembles that of ethanol-intoxicated worms (Davies et al., 2003; Bettinger and Davies, 2014). In *Drosophila melanogaster*, BK channels have been shown to play a central role in the development of drug tolerance to ethanol-induced sedation and dependence (Ghezzi et al., 2004, 2010; Cowmeadow et al., 2005, 2006). The literature on the role of slo channels in alcohol-altered behavior is discussed by Bettinger and Davies in this volume (2014). In conclusion, neuronal BK channels are considered as one of the central players in behavioral responses to ethanol observed across non-vertebrate and vertebrate species.

Ethanol-induced BK channel activation has been proposed as a mechanism for the neuroprotective effect of ethanol preconditioning against post-ischemic neuronal injury in mice (Wang et al., 2010). In contrast to BK channel activation, ethanol-induced BK channel inhibition in both rats and mice has been demonstrated to play a central role in ethanol-induced cerebral artery constriction (Liu et al., 2004; Bukiya et al., 2009). Likewise, this drug action has been hypothesized to also contribute to ethanol-induced aortic constriction (Walters et al., 2000). Exposure of human endothelial umbilical vein cells to 10–50 mM ethanol, however, leads to BK current potentiation, an ethanol action that leads to increased NO production and cell proliferation with eventual bolstering of endothelial function (Kuhlmann et al., 2004). Finally, a recent study shows that BK channel activation plays a critical role in alcohol-induced relaxation of guinea pig urinary bladder smooth muscle (Malysz et al., 2014).

In synthesis, in most neuronal tissues from mammals ethanol-induced activation of BK channels leads to decreased cell excitability whereas in vascular smooth muscle, ethanol-induced inhibition of BK channels leads to arterial constriction.

## Molecular targets and mechanisms that determine the final response in BK channel activity to acute ethanol

**Figure 1:**
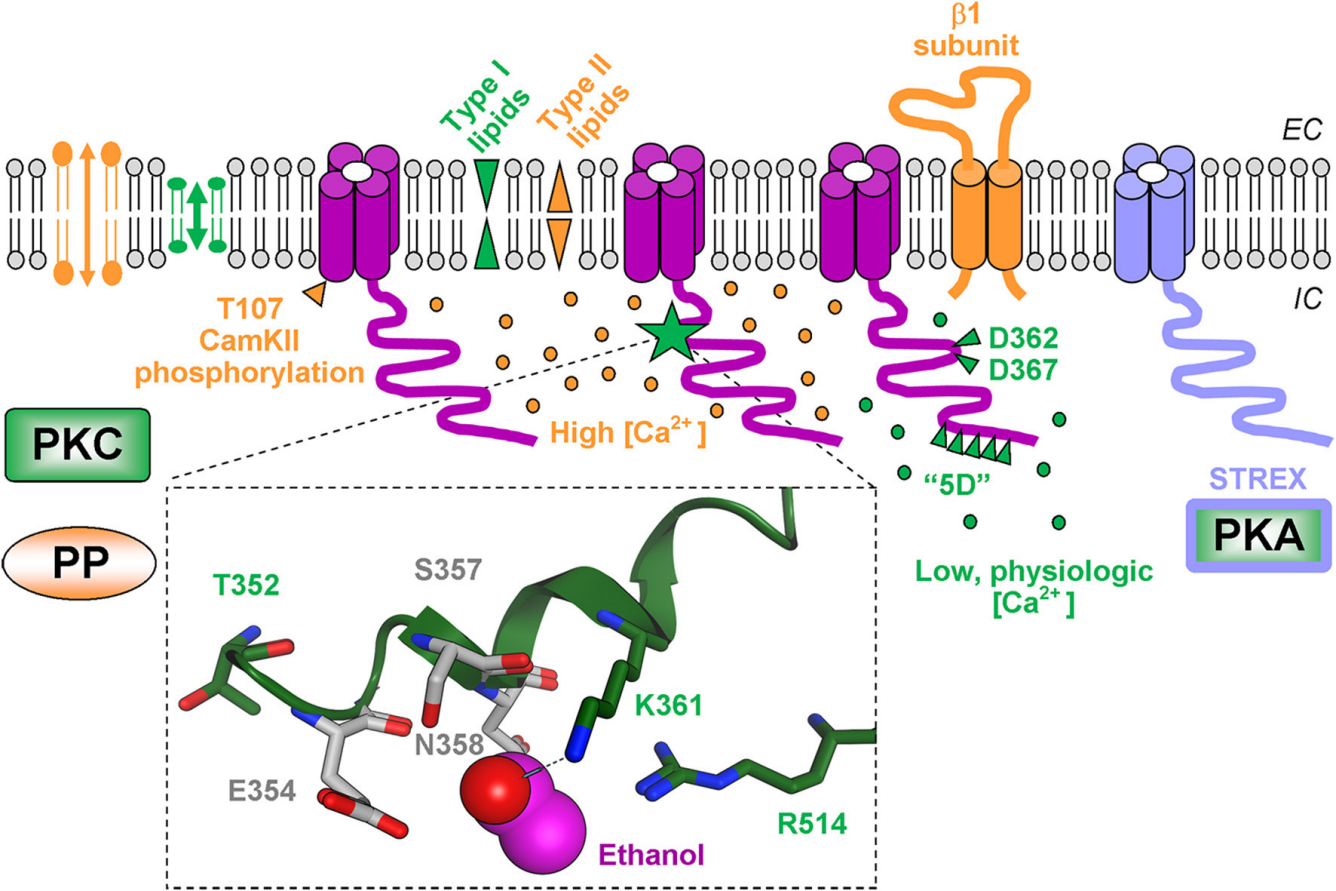
**Molecular determinants of ethanol final effect on BK channel activity following brief (up to several minutes) ethanol delivery to alcohol-naïve systems**. Functional BK channels are shown as tetramers. For clarity, however, the cytosolic tail domain (CTD) of only one α subunit within each tetrameric complex is displayed. Molecular components that favor ethanol-induced BK channel activation are shown in green whereas factors that counteract ethanol-induced BK channel activation are in orange. Insert depicts a recently identified ethanol-sensing site in the slo1 CTD. Ethanol molecule is depicted in pink; hydrogen bond between ethanol and K361 is highlighted by a light-blue dash line. Oxygen atoms are shown in red, nitrogen atoms are in blue.

Ethanol modulation of BK channel activity has been consistently reported in membrane patches that expressed either native or recombinant channel proteins and after reconstitution of channel subunits into artificial planar lipid bilayers of simple composition (Chu et al., 1998; Crowley et al., 2003, 2005; Yuan et al., 2008, 2011; Bukiya et al., 2011). Thus, functional targets of ethanol action are limited to the channel subunit themselves, their surrounding lipids and any possible interface. In a most reductionist approach, ethanol potentiation of hslo1 channels (from human brain) was observed with homomeric recombinant channel reconstituted into a single species phosphoglyceride, i.e., 1-palmitoyl-2-oleoyl-sn-glycero-3-phosphoethanolamine (POPE) (Crowley et al., 2003) indicating that this extremely simple proteolipid system must include an ethanol sensor(s). A summary of the different molecular factors and mechanisms that determine the Bk channel response to acute ethanol in alcohol-naïve systems in given in Figure 1.

**a) Identification of a Protein Pocket of Discrete Dimensions in the Slo1 Protein that Interacts with Ethanol thus Leading to Increased Channel Activity**

Slo1 channel proteins are conceptualized as “modular proteins,” i.e., with rather defined motifs each serving a defined channel function. Thus, slo1 proteins share with other members of the six transmembrane (TM6) voltage-gated superfamily of ion channels a TM “core,” which includes the voltage-sensing domain and the ion permeation pore-gate domain (Toro et al., 1998; Wang and Sigworth, 2009; Lee and Cui, 2010). In addition, BK channels include an additional segment (S) termed “0” leading to an exofacial N-end (Toro et al., 1998) and a long cytosolic tail domain (CTD), which includes two RCK domains largely responsible for sensing changes in physiological levels of Ca^2+^_i_ (Latorre and Brauchi, 2006; Lee and Cui, 2010; Hoshi et al., 2013). Remarkably, purely voltage-gated TM6 K^+^ (K_V_) channels are resistant to potentiation by ≤100 mM ethanol, these channels lacking the Ca^2+^_i_-sensing CTD that is found in slo1 proteins. As mentioned above, ethanol activation of slo1 channels has been linked to modulation of Ca^2+^-driven gating. Moreover, when studied in the same expression system, Ca^2+^_i_-sensitive slo1 is activated by ethanol while Na^+^-gated slo2 and H^+^-gated slo3, no matter the concentration of activating ion, remain ethanol-resistant. In addition, the S0-lacking but Ca^2+^_i_-sensitive TM2 K^+^ channel from *Methanobacterium thermoautotrophicum* (MthK) retains ethanol-sensitivity (Liu et al., 2013). Thus, it has been hypothesized that Ca^2+^_i_-sensing domains within the CTD, whether attached to a TM2 or TM6 core, are responsible for ethanol-sensing. Next, amino acid sequences of Ca^2+^_i_-sensing (e.g., cytosolic) regions of mslo1 and MthK were aligned to render regions that share sequence similarity (Bukiya et al., 2014). Computational modeling, point-mutagenesis and patch-clamp studies on mslo1 expressed in *Xenopus* oocytes revealed details of the ethanol-sensing site. The latter consists of several key elements (Figure 1): (1) K361 forms hydrogen-bond with ethanol molecule; (2) R514 provides net positive charge in the vicinity of the ethanol-K361 interaction point; (3) E354, S357, and N358 are located in close vicinity to ethanol, allowing access of ethanol to its K361 bonding partner. In a more recent work substitution of T352 with Ile resulted in elimination of ethanol-induced potentiation of BK current (Davis et al., 2014). T352 is located in the vicinity of the site recently described by Bukiya et al. (2014). Computational modeling shows that T352 points away from the ethanol-sensing pocket. Thus, T352I is unlikely to provide steric hindrance for hydrogen-bonding between ethanol and K361, the latter being critical for channel activation by ethanol. This hydrogen bonding, however, is hampered as the ethanol molecule cannot be positioned within the ethanol-sensing pocket when T352 is substituted by Ile (Bukiya and Dopico, unpublished). The critical role of T352 in ethanol sensing could be explained by the strategic position of this amino acid at the N-terminus of an α-helix within the slo1 CTD; polar/charged amino acids at the N- or C-terminus may neutralize the dipole moment associated with α-helix back-bone. Thus, in the T352I-substituted CTD, a neutral Ile could potentially disrupt the electrostatic interaction that likely exists between the polar Thr and the α-helix dipole. Eventually, the ethanol molecule cannot be positioned inside the ethanol-sensing pocket *possibly* due to repulsive electrostatic force(s) introduced by modification in the α-helix dipole moment. This explanation is in line with earlier speculation on the critical role of electrostatic interactions in the binding of polar molecules (including ethanol) to α-helical structures (Dwyer and Bradley, 2000).

Identification of ethanol-sensing site allows us to explain why BK channels fail to respond to ethanol in virtual absence of Ca^2+^_i_. Crystallographic data demonstrate that CTD conformation in Ca^2+^_i_-free environment (Wu et al., 2010) differs from that in presence of Ca^2+^_i_ (Yuan et al., 2010). As a result, in Ca^2+^_i_-free environment ethanol is no longer able to establish a hydrogen bond with K361 due to steric hindrance and repositioning of R514 away from the ethanol-sensing site (Bukiya et al., 2014). In addition to explaining the Ca^2+^_i_-dependence of ethanol action on BK channels, the identification of the ethanol-sensing site allows us to explain the “cut-off” phenomenon reported for 1-alkanol effects of BK channels originally described over a decade ago. This phenomenon shows BK current potentiation by propanol, butanol, pentanol, hexanol and heptanol, but refractoriness to octanol and nonanol (Chu and Treistman, 1997). Indeed, recent data show that the ethanol-sensing site in the slo1 CTS can accommodate 1-alkanols (propanol-heptanol) that activate BK channels but is unable to fit 1-alkanols that are ineffective (octanol and nonanol) (Bukiya et al., 2014). Thus, 20 years after the first report on the ethanol sensitivity of BK channels (Dopico et al., 1994), an ethanol-recognition site of discrete dimensions and drug-receptor interacting bonds responsible for ethanol activation of this channel have been identified in the channel-forming slo1 protein CTD (Bukiya et al., 2014). This ethanol-recognition pocket is close but does not significantly overlap with the slo1 protein CTD sites that sense Ca^2+^_i_. Thus, ethanol and Ca^2+^_i_ constitute heterotropic ligands of the BK channel.

**b) Slo Isoforms and their Regulation by Epigenetic Mechanisms following Protracted Ethanol Exposure**

Studies on recombinant homomeric slo1 channels cloned from a wide variety of mammalian species (human, rat and mouse brain) consistently document increased Po upon brief exposure to ethanol in alcohol-naïve systems (reviewed in Brodie et al., 2007; Mulholland et al., 2009). A notable exception is the bslo1 channel (cloned from bovine aortic smooth muscle (Dopico, 2003; Liu et al., 2003), this difference most likely being determined by Ca^2+^/Calmodulin-dependent protein kinase II (CamKII) phosphorylation of bslo1 at a residue that is not found in most slo1 isoforms (see separate section below).

Although slo1 proteins are products of a single gene (see above), pre-mRNA alternative splicing is a major source for diversity of BK channel proteins (Johnson et al., 2011; Kyle and Braun, 2014). In particular, BK-STREX is a stress-induced splice variant of BK channels that presents a phenotype associated with enhanced repetitive firing in neurosecretory cells (Xie and McCobb, 1998). The pituitary hormone-releasing cell lines GH3, GH4/C1, and GH4/C1-STREX have been used as models to address ethanol action on three BK channel subtypes that differ in slo1 subunits. In outside-out patches, however, 30 mM ethanol added to bath solution increases the steady-state activity of all three BK channel variants (Brodie et al., 2007).

In contrast to the rather homogeneous ethanol responses described in the previous paragraph, ethanol responses of BK channels vary greatly following “chronic” (hours) ethanol exposure, which involves “adaptation” of slo1 isoforms at a variety of levels. In two mammalian brain regions important in alcohol abuse and addiction, the supraoptic nucleus and the striatum, BK currents develop “tolerance” to ethanol (Knott et al., 2002; Pietrzykowski et al., 2004). A detailed study in the neurohypophyseal system shows that BK channel tolerance to ethanol exposure initially manifests itself as a slow-developing de-clustering within groups of channels and their subsequent internalization from the plasma membrane. After 24-h ethanol exposure, BK channels in the membrane are less clustered and less dense within those clusters (Pietrzykowski et al., 2004). Importantly, remaining BK channels display an almost complete lack of sensitivity to ethanol when acutely challenged again following withdrawal of the drug. The time-course of the acute ethanol response of native BK channels has been replicated in a study using hslo1 channels reconstituted into artificial lipid bilayers (Yuan et al., 2008). Collectively, these results indicate that, as interpreted for the immediate drug response of the naïve system, the time-dependent component of the channel response to acute ethanol is mainly determined by the channel-forming subunit itself and/or its immediate proteolipid environment.

To understand how the BK channels that were not internalized following several hrs-long ethanol exposure lost their ethanol sensitivity, analysis of BK channel properties in primary striatal cultured and HEK293 cells reveals that slo1 subunit expression is drastically altered by ethanol exposure. While the slo1 subunit is mostly the product of mRNAs coding for an ethanol-sensitive isoform in alcohol-naïve neurons, following chronic exposure it rapidly switches to an alcohol-insensitive variant called STREX (Pietrzykowski et al., 2008; Velázquez-Marrero et al., 2011). Of eight slo1 variants identified in primary striatal cultures, chronic ethanol led to elimination of variants more sensitive to ethanol while sparing those exhibiting much lower sensitivity to the drug (Pietrzykowski et al., 2008). This loss of ethanol-sensitive isoforms occurs because chronic ethanol exposure up-regulates a particular microRNA (miR9), which is a key factor controlling the expression of mRNA splice variants of slo1 channels. To further understand this phenomenon, authors focused on the slo1 channel mRNA 3′ untranslated region (UTR), which is known for its regulation of mRNA stability and being a target of miRNAs. The slo1 channel contains 3 distinct 3′ UTRs regions, each exhibiting different miRNA-binding patterns. Furthermore, the 3′ UTR containing a miR9-binding site is “stitched” to mRNA transcripts encoding slo1 isoforms with a high sensitivity to ethanol. Thus, it seems that chronic ethanol increases the probability of interaction between miR9 and its binding site located on specific 3′ UTRs by upregulating miR9. As a consequence of this interaction, mRNAs associated with these 3′ UTRs are degraded, eventually shifting the ratio of ethanol-sensitive/ethanol-tolerant variants leading to alcohol-resistance. Collectively, these data point to a central role for miR9 in ethanol action on striatal neurons and strongly suggest that increase in miR9 might contribute to development of tolerance to protracted ethanol challenge.

**c) BK β Subunits**

In most mammalian tissues, slo1 channels are associated with a variety of regulatory proteins, including the so called BK β subunits (types 1–4, encoded by *KCNMB1-4*, respectively). Remarkably, BK β types present a rather selective expression, with β1 and β4 being primarily abundant in smooth muscle cells and central neurons, respectively (Orio et al., 2002). These subunits substantially alter the ethanol effect on BK channels. For instance, the presence of β1 or β4 subunits may reduce acute ethanol potentiation of hslo1 after co-expression in human embryonic kidney (HEK) cells *via* an unknown mechanism that seems to be Ca^2+^_i_-independent (Feinberg-Zadek and Treistman, 2007). At physiological Ca^2+^_i_, however, the apparent Ca^2+^_i_-sensitivity of slo1 channels is drastically increased by β1 subunits with β4 failing to do so (Brenner et al., 2000). After cloning slo1 (cbv1) and β1 subunits from rat cerebrovascular myocytes, Dopico et al., found that β1 subunits shift the “crossover” for ethanol-induced channel activation to inhibition toward lower Ca^2+^_i_ (≤3 μM) (Bukiya et al., 2009). In contrast, β4 fails to modify such crossover when co-expressed with slo1 (Liu et al., 2008). BK β1 tuning of ethanol action results in ethanol inhibition of recombinant BK channels at low micromolar Ca^2+^_i_ (Bukiya et al., 2009), as found with the native cerebrovascular channel (Liu et al., 2004). Consistently with a key role for β1 in blunting slo1 channel activation by ethanol and favoring drug-induced inhibition, ethanol fails to activate native cerebral artery BK channels in *KCNMB1* knockout mice (Bukiya et al., 2009). Whether using native BK channels in freshly isolated mouse cerebral artery myocytes or recombinant BK proteins cloned from rat cerebral artery myocytes, β1 subunits inhibit BK channels at physiological Ca^2+^_i_ provided that critical levels of cholesterol are kept in the plasmalemma (Bukiya et al., 2009, 2011) (see Section on Membrane Lipids below). These studies led to the idea that Ca^2+^_i_, membrane cholesterol and BK β1 subunits conform a functional triad that determines the slo1 channel response to brief ethanol exposure (Bukiya et al., 2011).

**Figure 2:**
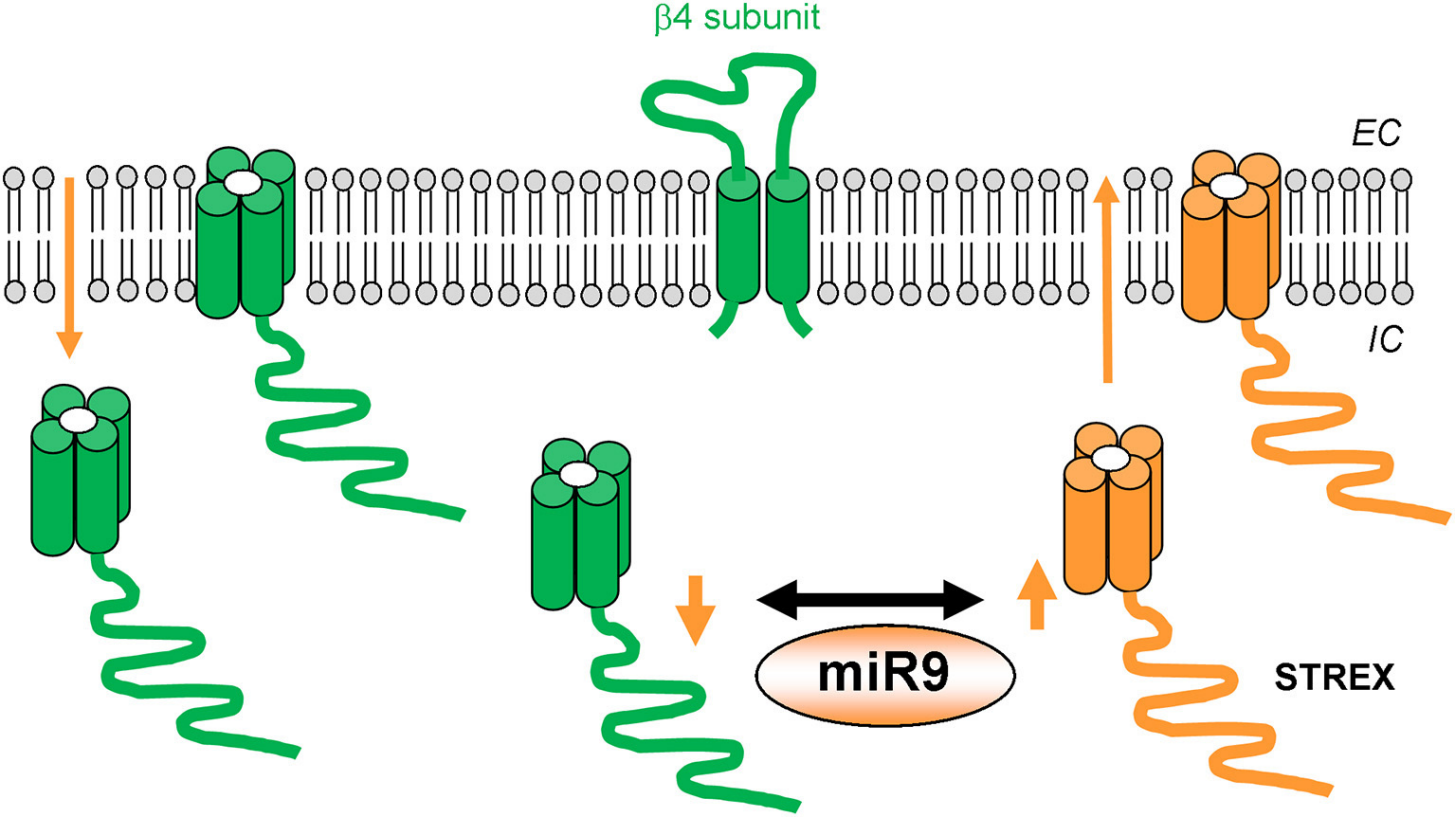
**Molecular factors that contribute to BK channel resistance to ethanol action following protracted (hours-long) alcohol exposure**. BK channel α isoforms and β4 subunits, which sustain ethanol-induced channel activation, are shown in green. BK channel α isoforms and mechanisms leading to ethanol “tolerance” are shown in orange.

When considering rat supraoptic magnocellular neurons, ethanol causes robust and mild activation of BK channels in nerve terminals and somata, respectively (Dopico et al., 1999b; Wynne et al., 2009). Likewise, in rat nucleus accumbens MSN, ethanol evokes robust and mild channel activation in somata and dendrites, respectively (Martin et al., 2004). Thus, in both supraoptic magnocellular and nucleus accumbens MSN neurons, BK isochannels from two domains within a same neuronal type display phenotypes consistent with differential expression of accessory channel β subunits within each domain. Moreover, single-channel recordings of BK activity in the MSN somatic region reveal biophysical properties consistent with co-expression of slo1 and β4 subunits. In contrast, similar electrophysiological recordings in the dendritic region, unveil a phenotype that is consistent with slo1 and β1 co-expression. Remarkably, while MSN somatic BK Po is significantly enhanced by 10–50 mM ethanol, their dendritic counterparts are ethanol-resistant. These data underscore the role of BK β1 in blunting ethanol-induced potentiation of BK channels (Martin et al., 2008). This study documents that homomeric slo1 and heteromeric hslo1 + β4 show similar ethanol sensitivity, as communicated by Liu et al. (2008) with mslo ± β4, yet in contrast to data from Feinberg-Zadek and Treistman (2007). Homomeric slo1 channels, however, rapidly develop tolerance to ethanol. In contrast, the ethanol-induced potentiation of hslo1 + β4 heteromers persists during the whole exposure to the drug, whether evaluated in heterologous expression systems or in freshly dissociated nucleus accumbens MSNs (Martin et al., 2008). Collectively, the studies described in this section indicate that BK β subunits can drastically influence both the BK channel's response to acute ethanol exposure in alcohol-naïve systems and the channel's response to ethanol following protracted drug administration. A summary of molecular entities and mechanisms participating in the BK channel response to protracted ethanol exposure is given in Figure 2.

**d) Phosphorylation of BK Channels and/or Channel-associated Proteins**

Ethanol-induced slo1 channel activation is also controlled by phosphorylation/dephosphorylation processes. This phenomenon was first reported in GH3 cells where potentiation of BK channel activity by 30 mM ethanol was blocked by protein kinase C (PKC) inhibition and favored by phosphatase (PP) inhibitors (Jakab et al., 1997). In GH4/C1 and GH4/C1-STREX cells, BK steady-state activity is increased only in some of the membrane patches under investigation, this variability being attributed to post-translational modification of slo1 proteins. PKC blockers diminish ethanol potentiation of BK channel activity in GH4/C1 cells but have no effect on GH4/C1-STREX cells. BK-STREX channel activation by ethanol, however, is protein kinase A (PKA)-dependent (reviewed in Brodie et al., 2007).

As mentioned in a previous section, bovine aortic BK channels (Walters et al., 2000) are inhibited by ethanol. It is highly likely that part of this drug effect is explained by the abundant expression of β1 subunits in vascular smooth muscle, as these subunits are responsible for blunting ethanol-induced potentiation and favoring inhibition of BK channel activity (see above). However, in contrast to other slo1 channels cloned from mammalian tissues, bslo1 (cloned from bovine aorta) is also inhibited by 10–100 mM ethanol (Dopico, 2003; Liu et al., 2003). This slo1 isoform distinctly includes a T107 in the S0-S1 intracellular loop. Incremental CaMKII-mediated phosphorylation of channel subunits at position 107 in the BK tetramer progressively increases channel Po and gradually switches the channel's ethanol responses from robust activation to inhibition. Thus, CaMKII phosphorylation of bslo1 T107 works as a “molecular dimmer switch,” this mechanism being able to override ethanol allosteric coupling to channel activation by physiological levels of Ca^2+^_i_. Notably, T107 is a region that is missing in K_V_ channels other than BK. Moreover, T107 equivalent position in mslo1, hslo1 and cbv1 is occupied by non-phosphorylatable residues, all these proteins forming homotetramers that are ethanol-sensitive (see above).

In a very recent study, Velàzquez-Marrero et al. (2014) examined the influence of protein kinase A (PKA), CaMKII, and PP on ethanol actions on slo1 ± β4 channels in HEK 293 cells and nucleus accumbens MSNs. Data show that the presence of β4 drastically alters the effects of PKA, CaMKII, and PP, echoing a study in HEK293 cells showing that this auxiliary subunit alters cAMP-mediated activation of BK channels (Petrik and Brenner, 2007). Interestingly, slo1 channel's rapid tolerance to ethanol is reversed following PP inhibition. In addition, slo1 + β4 channels develop ethanol tolerance in presence of CaMKIIN, a specific CaMKII inhibitor (Velàzquez-Marrero et al., 2014). Thus, while early studies focused on addressing the modulation of acute ethanol action on BK channel-forming proteins by kinases and phosphatases in alcohol-naïve systems, more recent studies are beginning to unveil a complex interplay between phosphorylation/dephosphorylation processes, slo1 proteins and the different types of accessory β subunits.

**e) Membrane Lipids**

Several studies documented a critical role for membrane lipids in tuning ethanol's final effect on BK Po. A consistent finding is a role for lipid “effective shape,” independently of lipid head net charge: for instance, ethanol-induced activation of hslo1 channels incorporated into planar lipid bilayers is favored by type I lipids, that is, those with a polar head cross sectional area larger than the tail area (e.g., phosphatidylserine), which introduce “positive monolayer curvature.” Conversely such ethanol action is blunted by type II lipids, that is, those with a polar head cross sectional area smaller than the hydrophobic tails/rings (e.g., phosphatidyglycerol, cholesterol) (Crowley et al., 2005). Considering that ethanol can be likened to a type I molecule, authors speculated that the reduced modulation of ethanol action by cholesterol in a POPE bilayer was due to a reduced action of a type II lipid (cholesterol) in a type II lipid environment (POPE). Cholesterol antagonism of slo1 channel activation by ethanol, however, has been attributed to a variety of factors. For example, cholesterol insertion in a bilayer favors liquid-order phase formation, which might facilitate ethanol partition in the bilayer and access to the channel target (discussed in Crowley et al., 2003). However, cholesterol is likely to modify ethanol action on dwell-times distribution and thus Po. Two non-mutually exclusive explanations for this antagonism on Po include opposite modification of physical bilayer properties by each modulator and direct protein-ligand interactions between modulator and the slo1 protein (reviewed in Dopico et al., 2012). Heteromeric BK channels composed of pore-forming cbv1 and β1 subunits cloned from rat cerebral artery myocytes are resistant to 50 mM ethanol when evaluated in cholesterol-free bilayers. Inclusion of 23 mol% cholesterol into the lipid mixture results in ethanol-induced BK channel inhibition (Bukiya et al., 2011). Although the molecular underpinnings of ethanol-cholesterol interactions in β1 subunit-containing BK channels remain unknown, there is a common theme from studies in artificial bilayers: cholesterol presence shifts the ethanol-exposed system toward lower Po, whether turning refractoriness into channel inhibition in the case of β1 subunit-containing BK channels or by diminishing ethanol-induced activation of homomeric slo1 channels. Remarkably, ethanol-cholesterol antagonism on slo1 channels could not be observed when cholesterol was substituted by *ent*-cholesterol, that is, its “mirror image” enantiomer (Yuan et al., 2011), suggesting that cholesterol tuning of the ethanol effect involves specific cholesterol-protein interactions. Indeed, both ethanol-recognition (see above) and cholesterol-recognition sites have been mapped to the slo1 CTD. The latter seem to include seven CRAC domains, with CRAC4 (the domain adjacent to the inner membrane leaflet where cholesterol is abundant) playing a major role (Singh et al., 2012). While cholesterol-recognition and ethanol-recognition sites on slo1 are nearby, they do not share key residues that are involved in recognition of each ligand.

Data on cholesterol-ethanol interactions on BK channels acquire particular relevance because these channels cluster in cholesterol-enriched rafts, and changes in cholesterol levels and distribution have been reported in cell membranes following chronic ethanol exposure (discussed in Crowley et al., 2003; Yuan et al., 2008). Indeed, work with lipid bilayer provides clear evidence that the membrane lipid composition influences tolerance to ethanol exposure. Thus, acute ethanol tolerance is observed in stable (20:1) phosphatidylcholine–dioleoylphosphatidylethanolamine (PC–DOPE) but not in sphingomyelin–DOPE bilayers (Yuan et al., 2008). It has been hypothesized that changes in the channel's lipid environment selectively alter ethanol access to sites in the channel protein that mediate opposing effects (potentiation vs. inhibition) on BK steady-state activity (Yuan et al., 2008).

An intriguing finding is that the ethanol response and adaptation of BK channels is sensitive to the bilayer thickness (Yuan et al., 2008), which can have particular importance in light of evidence for the presence of lipid rafts and location of BK channels within these domains (Weaver et al., 2007). Altering the thickness of the bilayer by adjusting the acyl chain length of the component lipids affects the time course of the acute response to alcohol and can turn ethanol-induced potentiation into inhibition. Hslo1 channels embedded in a thin bilayer are strongly potentiated by the drug whereas channels placed in a thicker bilayer are inhibited (Yuan et al., 2008). Insights into the mechanisms by which bilayer thickness affects BK function and pharmacology are becoming more accessible from our growing understanding of channel protein structure. In BK, the linker that connects the S6 gate to the RCK domains forms a passive spring with the gating ring and is involved in Ca^2+^_i_-dependent activation (Niu et al., 2004), the latter being required for ethanol potentiation of slo1 channels (Liu et al., 2008, 2013). A simple mechanical model to explain modulation of channel function by bilayer thickness has been hypothesized, in which lateral stress within the lipid bilayer in combination with forces generated by local hydrophobic mismatch between membrane lipids and the slo1 protein play a major role (Yuan et al., 2007). In synthesis, membrane lipid modulation of ethanol action on BK channel proteins may potentially result from lipid-induced modification of ethanol partition into the bilayer and access to ethanol's channel target site(s), modulation of bilayer physical properties by ethanol and lipid resulting in modification of channel gating, binding of ethanol and lipid species to distinct BK channel complex protein sites, which also would lead to gating modification, or any combination of these possibilities. Recognition sites in BK proteins have been only identified for a few lipid species (Dopico and Bukiya, 2014), and their role in ethanol modulation of channel function remains to be determined.

**f) Coupling to Nearby Ion Channels**

As mentioned above, BK channels cluster in membrane rafts that co-segregate signaling molecules and ion channels in addition to BK themselves. Thus, in most excitable tissues, BK channels constitute functional complexes, as first reported for BK and voltage-dependent Ca^2+^ channels (Marrion and Tavalin, 1998). Ethanol modulation of other ion channels may impact on the levels of Ca^2+^_i_ faced in the vicinity of the BK channels, activating Ca^2+^_i_ representing a key factor for ethanol to modulate BK currents (see above). In GH4/C1 cells, ethanol increases overall Ca^2+^_i_ in absence of extracellular Ca^2+^, an ethanol action that may contribute to drug modulation of BK channels (Jakab et al., 2006). Cross-talking between BK channels and nearby ion channels has been well studied in cerebrovascular smooth muscle where BK channel activity negatively feeds back on contraction driven by voltage-dependent Ca^2+^ influx. Contraction is also favored by IP3-sensitive, internal Ca^2+^-release channels that generate “Ca^2+^-waves.” In contrast, Ca^2+^-release via ryanodine-sensitive receptors (RyR) generates localized, “Ca^2+^-sparks,” which are located in close vicinity of and activate the BK channel, favoring vascular smooth muscle dilation (Jaggar et al., 1998; Narayanan et al., 2012). In cerebral artery smooth muscle cells, 50 mM ethanol fails to significantly modify Ca^2+^-waves and voltage-dependent Ca^2+^ currents. In sharp contrast, 50 mM blunts Ca^2+^ spark frequency and amplitude, a major mechanism thought to contribute to ethanol inhibition of BK currents, this ethanol action being responsible for cerebrovascular constriction (Liu et al., 2004). A recent study documents that both Ca^2+^ sparks and recombinant RyR2 (the type prevalent in rat cerebral artery myocytes; Vaithianathan et al., 2010) are inhibited by ethanol with an IC_50_ ~ 10 mM (Ye et al., 2014).

Conversely, ethanol modulation of BK currents may alter function of nearby ion channel proteins. In neurons, BK activation could also alter the refractory period of nearby voltage-dependent channels, leading to an actual increase in neuronal excitability (Warbington et al., 1996; Van Goor et al., 2001). This mechanism has been advanced to explain slo-mediated tolerance to the sedative/hypnotic effect of alcohol in *drosophila* (Ghezzi and Atkinson, 2011). Studies of ethanol action on the fly have been comprehensively reviewed in this volume by Bettinger and Davies (2014). In synthesis, ethanol actions on BK currents are usually a composite that results from drug action on BK channel complex themselves and on other ion channel proteins that modulate BK channel activity, making it extremely difficult to extrapolate ethanol effects on BK channels reported in specific cells or cell domains to another.

## Concluding remarks and future challenges

Data summarized and discussed in this review make evident that a multiplicity of molecular target and mechanisms conditions the final response of BK currents to acute ethanol exposure in alcohol-naïve systems, with current potentiation, refractoriness and inhibition all being reported, including within different domains of a given neuronal type. In addition, functional association between BK channels with other ion channels within a cell domain may determine that a given ethanol action on BK channels results in opposite effects. Ethanol activation of BK channels clearly reduces excitability in nucleus accumbens MSN, yet such drug action may actually increase excitability in *Drosophila* neurons as the refractory period of other voltage-gated conductances may be affected. Along the same lines, BK current potentiation and voltage-gated Ca^2+^-channel inhibition contribute to decrease neuropeptide release from neurohypophyseal axon terminals, yet BK channel activation in growth-hormone release cells cannot overcome drug action on intracellular channels and signaling, resulting in increased hormone release by alcohol. Thus, ethanol actions on BK channels in one system cannot be simply extrapolated to another. Molecular multiplicity leads to different ethanol responses even within different domains within a given neuronal type, as reported for supraoptic neurons and nucleus accumbens MSN. On a practical note, identification of the molecular entities and mechanisms that determine ethanol final effect on BK currents is critical for any therapeutic intervention to prevent or revert modification of BK channel-regulated physiology by acute ethanol exposure.

In light of the BK channel's sensitivity to ethanol intoxicating concentrations and the channel expression in regions central to the development of dependence to drugs of abuse (including ethanol itself), a number of studies have probed BK channel's role in behavioral tolerance. Moreover, several of the key elements that determine the final ethanol response of BK channels in alcohol-naïve systems (e.g., slo1 channel isoforms, BK β subunits, membrane lipids) also play a key role in a modified system response to protracted ethanol exposure. Plastic changes at the molecular, cellular and neurocircuitry levels very likely result in behavioral manifestations of ethanol misuse and consumption. Indeed, in a 2-bottle choice drinking paradigm, BK β1 and β4 subunits have opposite effects on voluntary alcohol intake of dependent rodents, with the former and the latter respectively accelerating and attenuating the escalation (Kreifeldt et al., 2013). Finally, it has been advanced that presence of enhanced acute behavioral tolerance to alcohol in humans can serve as a marker for the likelihood of future development of alcoholism (Schuckit, 1985a,b, 1994; Heath et al., 1999). Therefore, understanding the adaptations in neuronal BK currents and the underlying molecular mechanisms that sustain ethanol tolerance and dependence is of fundamental significance to gain insights on the bases of alcohol vulnerability, and even develop a molecular target identification-designed therapy for treating alcohol misuse.

### Conflict of interest statement

The authors declare that the research was conducted in the absence of any commercial or financial relationships that could be construed as a potential conflict of interest.

## Abbreviations

AMPA: α-amino-3-hydroxy-5-methyl-4-isoxazolepropionic acid
AP: action potential
BK: Ca^2+^ and voltage-gated, large conductance K^+^
cAMP: cyclic adenosine monophosphate
Ca^2+^_i_: cytosolic Ca^2+^; Ca^2+^-activated K^+^
CaMKII: Ca^2+^ /calmodulin-dependent protein kinase II
CNS: central nervous system
CTD: cytosolic tail domain
DRG: dorsal root ganglia
EC_max_: maximal effective concentration
EC_50_: half-maximal effective concentration
EPSP: excitatory post-synaptic potential
GH3, GH4/C1: rat pituitary epithelial-like tumor cell lines
HEK: human embryonic kidney (cell line)
IP3: Inositol trisphosphate
MSN: medium spiny neurons
MthK: potassium channel from *Methanobacterium thermoautotrophicum*
NMDA: N-methyl-D-aspartate
PKA: protein kinase A
PKC: protein kinase C
Po: single channel open probability
PP: phosphatase
RCK: regulator of conductance for K^+^
RyR: ryanodine receptor
S: transmembrane segment in ion channel core
SK: potassium channels of small conductance
TM: transmembrane domain.
